# Endothelial Progenitor Cells (EPCs) as Gene Carrier System for Rat Model of Human Glioma

**DOI:** 10.1371/journal.pone.0030310

**Published:** 2012-01-20

**Authors:** Nadimpalli Ravi S. Varma, Branislava Janic, A. S. M. Iskander, Adarsh Shankar, Mohammed P. I. Bhuiyan, Hamid Soltanian-Zadeh, Quan Jiang, Kenneth Barton, Meser M. Ali, Ali S. Arbab

**Affiliations:** Cellular and Molecular Imaging Laboratory, Department of Radiology, Henry Ford Hospital, Detroit, Michigan, United States of America; University of Texas, M.D. Anderson Cancer Center, United States of America

## Abstract

**Background:**

Due to their unique property to migrate to pathological lesions, stem cells are used as a delivery vehicle for therapeutic genes to tumors, especially for glioma. It is critically important to track the movement, localization, engraftment efficiency and functional capability or expression of transgenes of selected cell populations following transplantation. The purposes of this study were to investigate whether 1) intravenously administered, genetically transformed cord blood derived EPCs can carry human sodium iodide symporter (hNIS) to the sites of tumors in rat orthotopic model of human glioma and express transgene products, and 2) whether accumulation of these administered EPCs can be tracked by different *in vivo* imaging modalities.

**Methods and Results:**

Collected EPCs were cultured and transduced to carry hNIS. Cellular viability, differential capacity and Tc-99m uptake were determined. Five to ten million EPCs were intravenously administered and Tc-99-SPECT images were acquired on day 8, to determine the accumulation of EPCs and expression of transgenes (increase activity of Tc-99m) in the tumors. Immunohistochemistry was performed to determine endothelial cell markers and hNIS positive cells in the tumors. Transduced EPCs were also magnetically labeled and accumulation of cells was confirmed by MRI and histochemistry. SPECT analysis showed increased activity of Tc-99m in the tumors that received transduced EPCs, indicative of the expression of transgene (hNIS). Activity of Tc-99m in the tumors was also dependent on the number of administered transduced EPCs. MRI showed the accumulation of magnetically labeled EPCs. Immunohistochemical analysis showed iron and hNIS positive and, human CD31 and vWF positive cells in the tumors.

**Conclusion:**

EPC was able to carry and express hNIS in glioma following IV administration. SPECT detected migration of EPCs and expression of the hNIS gene. EPCs can be used as gene carrier/delivery system for glioma therapy as well as imaging probes.

## Introduction

Due to their unique property to migrate to pathological lesions, stem cells are considered to be used as a delivery vehicle for therapeutic genes to tumors, especially for glioma [1], [2]. Investigators have used neural and mesenchymal stem cells as vehicles for delivering cytotoxic or therapeutic genes [1], [3], [4]. These cells were administered either locally or systemically. Schichor et al. have pointed out that cells should meet the following criteria to be used as gene delivery vehicles in glioma: 1) cells should be used as an autologous transplant system in each glioma patient to avoid potential immune response, and 2) within human brain parenchyma, cells should exhibit active motility directed toward glioma tissues [4]. Endothelial progenitor cells (EPCs), a subpopulation of pluripotent hematopoietic stem cells (HSC), showed active migration and incorporation into neovasculature of glioma when administered locally or systemically [5], [6]. Based on EPCs' characteristics, it is possible to use these cells as carriers or delivery vehicles for therapeutic genes to tumors or glioma, which can be administered either systemically or locally. Moreover, these EPCs can be collected from patients' peripheral blood.

In order to evaluate efficacy and appropriateness of cell based therapy, it becomes critically important to track the movement, localization, engraftment efficiency, and functional capability or expression of transgenes of selected cell populations following transplantation. The available techniques are suboptimal in this regard. For instance, in vivo fluorescent or bioluminescent molecular and/or cellular imaging techniques lack the resolution necessary to localize the sites of active cell migration and accumulation. Although nuclear medicine techniques can be used to track the radioisotope tagged administered cells, associated radiation injury and short half-life of usable radioisotopes are their drawbacks. Recently, we have created superparamagnetic iron oxide (SPIO)-transfection agent complexes using two FDA approved agents; Ferumoxides (Fe) and Protamine sulfate (Pro), to label a broad range of mammalian cells. The labeled cells can then be used as probes to localize physiological or pathological processes using magnetic resonance imaging (MRI) for high-resolution images in a clinical setting [7], [8]. Cells labeled with the ferumoxides-protamine sulfate (FePro) complexes can be imaged at clinically relevant MRI fields using standard imaging techniques and also at higher fields typical for animal experiments. We and others have shown that labeling cells with ferumoxides did not alter viability and functional capability of cells or differential capacity of stem cells [9].

Human sodium iodide symporter (hNIS) is an intrinsic trans-membrane glycoprotein that mediates transport of iodide into the thyroid follicular cells [10], [11]. This transport system also transports Tc-99m pertechnetate (Tc-99m) that can be imaged by gamma camera [12], [13]. Visualization and quantification of Tc-99m activity at the site of interest (following administration of transduced cells) would provide the evidence of homing, viability, and expression of exogenous hNIS gene in transduced cells. The *in vivo* use of genetically modified EPC requires that duration and level of expression of the encoded transgene to be monitored. Ideally, the monitoring system should also be able to monitor cell homing and viability, and demonstrate the persistence of site-specific gene expression *in vivo*. MRI can be used with iron-based contrast to detect homing of administered stem cells with high resolution [5], [6] but MRI may not be useful to monitor the functional activity of the cells. Although MRI has been used to monitor the expression of ferritin in transduced implanted cells; expression of ferritin is not considered to be a target for treatment [14]. Expression of hNIS with resultant Tc-99m uptake can conveniently be imaged with single photon emission computed tomography (SPECT) or gamma camera that can determine hNIS expression *in vivo*, which will indicate the effectiveness of EPC to carry gene of interest to the sites of tumors.

The purposes of this study were to determine whether 1) intravenously administered genetically transformed cord blood derived EPC can carry hNIS to the sites of tumors in rat orthotopic model of human glioma and express transgene products, and 2) whether accumulation of these administered EPC can be tracked by *in vivo* MRI and the expression of hNIS can be determined by *in vivo* Tc-99m SPECT. Ultimate goal was to determine whether in future, EPC could be used as gene carrier/delivery system for glioma therapy.

## Materials and Methods

### Ethics statement

Human cord blood was collected under Henry Ford Health System Institutional Review Board (IRB) approved protocol (protocol number 3287). Collection of cord blood was anonymous and patients' profile was not documented. Ethics committee (IRB committee) approved waiver of consent. All production of lentivectors and transduction of stem cells were performed under Henry Ford Health System Institutional Recombinant DNA and Biosafety Committee (IRDBC) approved protocol (protocol number 2008.12). Animal experiments described in the manuscript were approved by our animal care and user committee at Henry Ford Health System (protocol number 753 and 988) according to the guideline and policies of office of laboratory animal welfare (OLAW) and public health service, National Institutes of Health. All the experiments were performed according to the approved protocol.

### CD133+ endothelial progenitor cells (EPCs)

Freshly collected and cultured CD34^+^/CD133^+^ EPCs were used for transduction and magnetic labeling. CD34^+^/CD133^+^ EPCs were collected from the human cord blood. In brief, cord blood mononuclear cell population was generated by Ficoll gradient centrifugation and was enriched for CD34^+^/CD133^+^ cells by immunomagnetic positive selection using the MidiMACS system (Miltenyi, Auburn, CA) according to the manufacturer's protocol. Freshly prepared CD34^+^/CD133^+^ cells were incubated in stem cell basal media supplemented with 40 ng/ml of stem cell factor (SCF), 40 ng/ml of fms-like tyrosine kinase receptor-3 ligand (FLT3L) and 10 ng/ml of thrombopoietin (TPO) (all from CellGenix, IL). Initially cells were suspended in media at 1×10^6^ per ml and grown in 5% CO_2_/95% air at 37°C in humidified atmosphere, with fresh media added every third day. The cells were propagated for 7–10 days. To confirm the purity of the culture isolated from cord blood, flowcytometry was done at day 1 to assess the levels of progenitor markers CD133, CD34, hematopoietic CD45, CD117 and CD14 markers, and CD20 and CD3 that are commonly expressed on B and T cells, respectively. More than 90% cells were CD34^+^/CD133^+^.

### Development of hNIS gene carrying lentivector

To construct pCDH-hNIS vector, the hNIS gene was amplified using NISCMVF1 and NISCMVR1 primers. The NISCMVF1 primer was a 27-mer forward primer with an XbaI site (5′ACT*TCTAG*A*G*CCACCATGGAGGCC-3′). NISCMVR1 was a 32-mer reverse primer with an EcoRI site (5′CGC*GAATTC*TCAGAGGTTTGTCTCCTGCTG-3′). The restriction enzyme recognition sites are in italics. A 25 µl polymer chain reaction (PCR) sample was prepared according to MBI Fermantas using pNIS vector (a gift from Dr. Barton) as a template. PCR was carried out using Mastercycler (Eppendorf, Germany), with following program: 95°C for 5 min to denature and then amplification was carried out over 30 cycles of 94°C for 1 min, 55°C for 1 min and 72°C for 1 min; and after the last cycle the reaction was held at 72°C for 10 min. Amplified *hNIS* gene was digested by *Xba*I and *Eco*RI, followed by ligation into the corresponding sites on plasmid plent-CMV (SBI, USA). The ligated DNA was transformed into E. coli cells by the heat-shock method. Ampicillin-resistant transformants were then selected, followed by plasmid extraction and restriction digestion analyses.

### Lentivirus production

293TN cells (SBI, USA) were seeded at a density of 1.6×10^8^ per 100 mm tissue culture dish and transiently transfected with lentivirus transfer vectors (pCDH-hNIS) and three viral packaging plasmids (pPACKH1-GAG, pPACKH1-Rev, pVSV-G) (SBI, USA) according to the manufacturer protocol. After 16 h the media was replaced with Dulbecco's modified essential medium (DMEM) supplemented with 10% fetal bovine serum (FBS) and 20 mM HEPES, pH 7.05 (Sigma). The supernatants were harvested at 48 and 72 hours and further concentrated using 5× PEG solutions (SBI, USA). Concentrated virus was stored at −80°c prior to use. Titers of concentrated lentiviral vector were determined using Global UltraRapid Lentiviral Titer Kit (SBI, USA). Lentivector titers were in the range of 10^9^ transducing units (TU)/ml.

### Optimization of viral dose

To determine the optimal viral dose, cells (1×10^6^ cells) were infected at 37°C, 5% CO_2_ with increasing dose of viral particles (1000, 2000 and 5000 viral particles per cell) in 200 µl. After one hour, 1 ml of fresh media was added and transferred to a 6-well plate and further incubated for 48 hours. Viability and transgene expression were determined by trypan blue dye exclusion test and Tc-99m uptake assay, respectively.

### Tc-99m uptake assay

To test Tc-99m uptake ability of transduced cells, 10 µCi of Tc-99m (Mallinckrodt, USA) was added to 1×10^6^ cells (in serum free media) and incubated at 37°C for 30 min. Following incubation, cells were washed twice with 5 ml of phosphate buffered saline (PBS) and cell associated Tc-99m activity in the resultant cell pellets was measured using gamma counter (Wizard 1420, PerkinElmer, USA). Tc-99m uptake was also determined following magnetic labeling of transduced EPC.

### Transduction of EPCs with lentiviral vector containing hNIS gene

**Figure 1:**
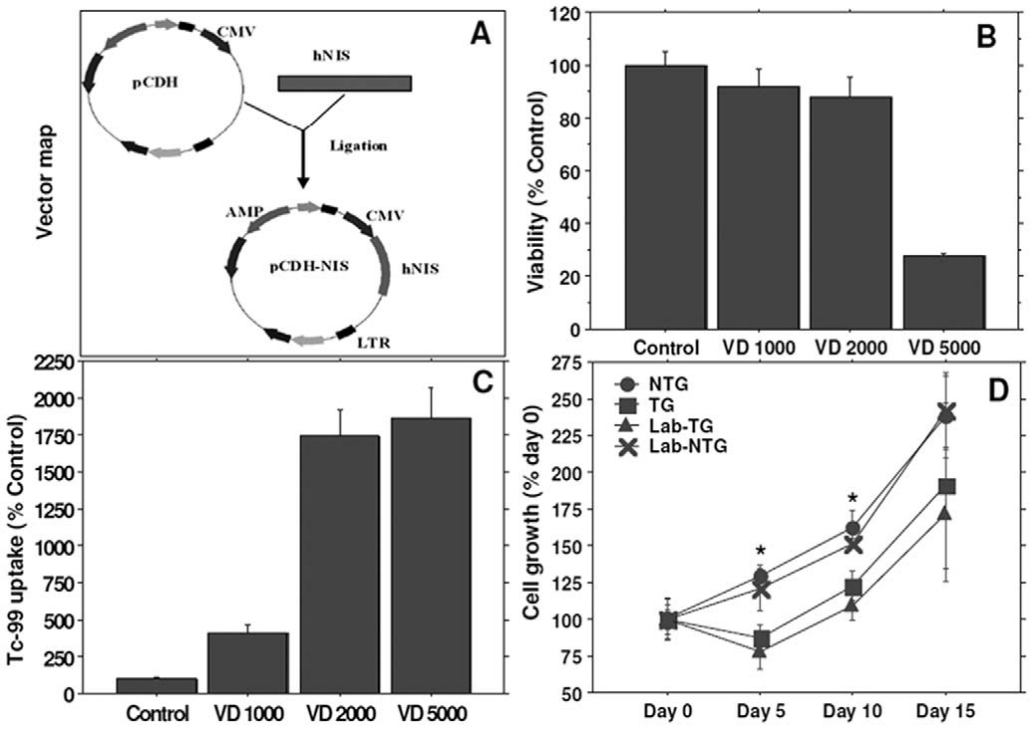
Vector design, Tc-99m uptake assay, viability and proliferation following FePro labeling. (**A**) Map of the lentivector carrying hNIS. Effect of viral doses (VD) on viability (**B**) and Tc-99m uptake (**C**) showed that at 5000 viral particles per cells only about 35% cells were viable, although increased Tc-99m activity was observed at this dose. However, based on viability and Tc-99m uptake subsequent experiments were performed with a dose of 2000 viral particles per cell. Magnetic labeling showed decreased proliferation on day 3 compared to unlabeled cells, however, proliferation rate recovered on later days (**D**). * = p<0.05.

Following optimization all subsequent transduction of EPC was performed by maintaining viral dose of 2000 particles per cell. Lentiviral vector carrying the hNIS gene was used to transduce cells. Expression of the gene product was under CMV (cytomegalovirus) promoter (**Figure 1A**). During transduction cells were maintained at a concentration of 10 million per ml for initial 1 hour and then diluted ×10 with complete stem cell media. Transduction efficiency was determined on day 3 and following magnetic labeling by Tc-99m uptake assay. These transgenic cells were magnetically labeled on day 4 after transduction using FePro.

### Labeling of cells with ferumoxides-protamine sulfate (FePro) complex

Cells were labeled according to our recently published updated method [8]. In brief, ferumoxides (Fe) (Feridex IV®, Berlex Laboratories, Inc, Wayne, New Jersey, 100 µg/ml) were directly added to the cell suspension in serum free media and then protamine sulfate (Pro) (American Pharmaceuticals Partner Inc. Schaumburg, IL, 3 µg/ml) was added. The FePro complexes were formed in the cell suspension. After 15 minutes of incubation in serum free media, an equal volume of complete media (containing different cytokines) was added to the cell suspension and further incubated for 4 hours. The cells were then collected, washed with sterile PBS and resuspended at the specific concentration for injection. Labeling efficiency and intracellular iron concentration were also determined as described in our previous reports [8], [15].

### Cellular viability, proliferation of Fe-Pro labeled and/or transduced EPCs

Cells were washed at least twice with sterile PBS and resuspended in PBS at the concentration of 3×10^7^ cells/ml. A small aliquot of cells was mixed with trypan blue dye and checked under a microscope to determine cell viability. Specific number of control (non-transduced non-FePro labeled), non-transduced FePro labeled, and transduced FePro labeled, cells were placed back into culture flasks and total numbers of cells in the cultures were monitored up to day 15.

### Differential capacity of FE-Pro labeled and/or transduced EPCs

Differentiation potential of EPCs transduced with lentivirus-hNIS (labeled or unlabeled) as well as control EPCs was assessed. EPCs were suspended in stem cell basal media supplemented with 2% fetal bovine serum (FBS) and 2 ng/ml of Vascular Endothelial Growth Factor (VEGF) and plated on glass cover slips coated with fibronectin at the concentration of 2×10^5^/cm^2^. Cells were allowed to differentiate for 2 weeks. Old media was replenished with fresh media on every 3 days and cells were monitored by microscopy for morphological changes associated with differentiation. After 2 weeks, differentiated transduced EPCs were analyzed by immunocytochemistry for the expression of endothelial cell specific markers. The cells were analyzed using endothelial specific antibodies such as mouse anti-human anti CD31, rabbit anti-human anti von Willebrand Factor (vWF) (DakoCytomation) and rabbit anti-human anti CD309 (VEGFR2 or KDR) (Thermo Scientifics). Texas Red or FITC conjugated secondary antibodies (Jackson ImmunoResearch, Inc) were used. Negative control samples were treated with secondary antibodies only. All the antibodies were used according to supplier's directions. To visualize nuclei, cells were counterstained with DAPI nuclear stain. Cells were analyzed by fluorescent microscopy. Fluorescent images were acquired with a fixed exposure time (10 sec for FITC and 20 sec for Texas Red labeled cells).

### Human glioma cells (U-251)

Human glioma cells (U-251, gift from Dr. Steve Brown, HFH) were cultured in 75 cm^2^ tissue culture flasks with Dulbecco's modified Eagle's medium (DMEM) supplemented with 10% FBS, penicillin (100 IU/mL), and streptomycin (100 µg/mL) until they were 80–90% confluent. Then, the cells were collected by trypsinization, washed and centrifuged to make a cell suspension of 4×10^5^cells/5 µl.

### Animal model

Athymic nude rats 6–8 weeks of age and 150–170 g of weight (Charles River Laboratory, Inc.) were anesthetized by intraperitoneal injection using ketamin/xylazine mixture (100 mg/kg ketamine, 10 mg/kg xylazine) and were placed on a stereotactic head holder. The tumor was implanted according to our reported methods [16]. In brief, after exposing the skull and drilling a hole 3 mm to the right and 1 mm anterior to the bregma, a 10 µL micro-syringe fitted with 26 s gauge-needle containing tumor cells (4×10^5^) in 5 µl was lowered to the depth of 4 mm, then raised to the depth of 3 mm. The U251 cells were injected stepwise at a rate of 0.5 µL/30 sec until the entire volume had been injected. During and after the injection, careful note was made of any reflux from the injection site. Two to three minutes after completing the injection the syringe was withdrawn in a stepwise manner. The surgical hole was sealed with a bone wax, cleaned, and the overlying skin was sutured. There were a total of four groups of animals. A total of 26 animals were included in this study; however, due to lack of optimal tumor size and missing SPECT studies 5 animals were excluded. There were at least 4 animals in each group (4–6 animals per group).

### Intravenous administration of hNIS transduced and FePro labeled EPCs

Five to ten million of EPCs were intravenously administered in rats bearing human glioma through tail vein 14 days after tumor implantation. All animals underwent pre-injection MRI. Four different groups of animals received different doses of cells as follows: 1) 5×10^6^ of FePro labeled non-transduced (LNTG) EPCs (5 animals as negative control for Tc-99m SPECT), 2) 5×10^6^ of non-FePro labeled transduced (NLTG) EPCs (4 animals, negative control for MRI), 3) 5×10^6^ of FePro labeled transduced (LTG) EPCs (6 animals), and 4) 5×10^6^ of FePro labeled transduced plus 5×10^6^ of non-FePro labeled transduced (LTG+NLTG) EPCs (6 animals). Different number of transduced EPCs (groups 2 or 3 vs 4) was used to determine whether higher number would allow better detection of transgene expression by Tc-99m SPECT. FePro labeled and unlabeled transduced EPCs (group 2 vs 3) were used to determine whether FePro labeling would decrease the migration and transgene expression in the tumors. Following administration of EPCs, rats underwent MRI and Tc-99m SPECT to determine the migration and accumulation of FePro labeled and transduced cells carrying hNIS, on days 7 and 8, respectively. Timing of MRI was based on our previous experiences where we have shown that most of the migration and incorporation of administered EPCs were dependent on the tumor size and varied from 3–14 days following IV administration [5], [6]. We have selected date of SPECT studies after MRI because the transportation of radioactive animals to other building was restricted by IACUC. Recently we have shown that transduced EPCs can express the transgene products even after 38 days [17].

### Magnetic Resonance Imaging (MRI) study

To prepare each rat for MRI studies, the rat was anesthetized with 1.5–2.0% isoflurane in oxygen. Rats were secured to a customized cradle. Rats were studied by MRI 1 day before and 7 days after IV administration of EPCs. MR images were obtained with a 3.0 Tesla clinical system (Signa Excite, GE health) using 50 mm diameter small animal imaging coil (Litzcage small animal imaging system, Doty Scientific Inc, Columbia, SC). Three dimensional (3D) isotropic FIESTA images were acquired. The following parameters were used to acquire the images. For isotropic 3D FIESTA images: TR = 11.4 ms, TE = 5.6 ms, using a 200×200 matrix, FOV = 60 mm, and NEX = 2, effective slice thickness was 0.3 mm.

### MR image analysis

Circular ROIs were drawn on the tumor sections encompassing as much tumor area as possible and signal intensity histogram profiles as well as average signal intensity were recorded. Animals from FePro labeled transduced (LTG) and FePro non-labeled transduced (NLTG) groups were included in the analysis.

### SPECT Study

Within 24 hours after last MRI, animals underwent SPECT studies using Tc-99m to determine the status of genetically transformed cells at the sites of tumors. Animals were anesthetized using ketamine/xylazine (100/10 mg/kg) and received 1 mCi of Tc-99m through tail vein injection in a volume of 1–2 ml. 60 minutes after Tc-99m injection, animals were wrapped with padded sheet to keep them warm and placed in the imaging holder under anesthesia. SPECT was acquired with a dedicated PRISM 3000 gamma camera fitted with mutli-pinhole rat collimator, 360 degree rotation with 36 degree increments, 180 sec per projection, using 256×256 matrices with a field of view of 4×6 cm. The total time required for acquiring SPECT was about 31 minutes.

### Euthanasia and histological analysis

Immediately after SPECT, animals were euthanized and perfused with saline and paraformaldehyde to dissect and further analyze tissues by histology. The animals were euthanized with 100 mg/kg of pentobarbital administration (intravenous or intraperitoneal). The radioactive fluids were collected and contained in a shielded area to decay. Whole brains were collected for histochemical determination of iron labeled cells using Prussian blue (PB) staining and endothelial cells markers such as CD31 and vWF using human specific antibodies (DakoCytomation, USA). Expression of hNIS in accumulated cells was determined by staining with anti-hNIS antibody (Genetex, TX, USA).

### Supplemental studies

To determine the distribution of IV administered EPCs in different organs at different time points, animals bearing glioma received 5 million In-111-oxine labeled EPCs and the animals underwent SPECT studies 1 to 72 hours following administration. To differentiate between the activity of In-111 and that of In-111 labeled cells on SPECT images, animals bearing glioma that received In-111-oxine alone also underwent SPECT studies.

### Data analysis

All data are expressed as mean ± standard deviation. The data were analyzed with two-way analysis of variance and the significance level was set at 0.05.

## Results

### Vector design, Tc-99m uptake assay, viability and proliferation following magnetic labeling

**Figure 1A** shows the vector design. We have used CMV as promoter for the expression of hNIS, based on our previous experiments, where we have used four different promoters (CMV, EF-1, UBC and SV40) [17]. We determined the successful transduction of cells by Tc-99m uptake. For optimizing the transduction efficiency without compromising the cell viability it is important to determine the cell to viral dose. Cells were incubated with different doses of viral particles and then the cell associated Tc-99m activity and cell viability were determined. Based on the viability and Tc-99m uptake, it was determined that the dose of 2000 viral particles per cells was optimal for transduction of EPCs (**Figure 1B, 1C**). We used this ratio in all subsequent studies.

Following transduction, EPCs were magnetically labeled using FePro and used as MRI probes to monitor the cell migration and accumulation at the sites of implanted glioma in rat brain. As described in our previous studies, we achieved labeling efficiency of almost 100% [8] that resulted in successful detection of iron labeled cells by *in vivo* MRI. However, it is also important to determine the proliferation capacity of transduced cells with or without FePro labeling. Following transduction of hNIS gene into EPCs, significant differences were observed (day 5 and 10) in the growth curve compared to that of non-transduced EPCs (**Figure 1D**). After labeling EPCs with FePro, the same difference was observed between non-transduced FePro labeled and hNIS transduced FePro labeled EPCs. However, there were no differences in cell viability determined by trypan blue dye exclusion test (data not shown) among control and transduced EPCs with or without FePro labeling. Altogether these results indicated that hNIS transduction may be the cause of transient growth retardation observed on days 5 and 10 in both non-labeled transduced and FePro labeled transduced EPCs (although similar pattern of growth curve was observed for all groups between days 5 to days 15). On the other hand, FePro labeled cells exhibited significant decrease in Tc-99m uptake (100±1.15% vs. 81.78±8.55%, p<0.02) in transduced EPCs.

### Differential capacity of transduced cells

**Figure 2:**
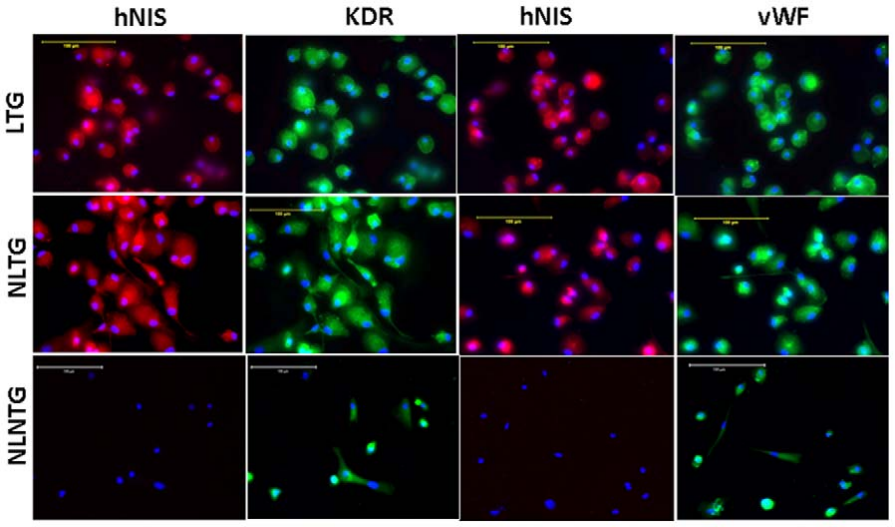
Differentiation capacity of transduced cells. Expression of endothelial markers (KDR and vWF) in FePro labeled transduced (LTG) or non-Fepro labeled transduced (NLTG) EPCs following incubation in endothelial cell differentiation media for 15 days. All of these cells also showed expression hNIS. None of the control EPCs showed expression of hNIS, although expression of both the markers of endothelial cell was observed.

To determine whether transduction of exogenous genes affected the ability of non-labeled and FePro-labeled EPCs to differentiate towards endothelial cell lineage; non-transduced non-FePro labeled (control), transduced non-FePro labeled, and transduced FePro labeled EPCs were cultured in endothelial cell differentiating media. After 15 days the expression of mature endothelial cell type markers, VEGFR2 (KDR) and vWF, was determined by immunocytochemistry. No differences were observed between control, transduced and transduced FePro labeled EPCs in their ability to express these endothelial markers (**Figure 2**).

### MRI and SPECT analysis

**Figure 3:**
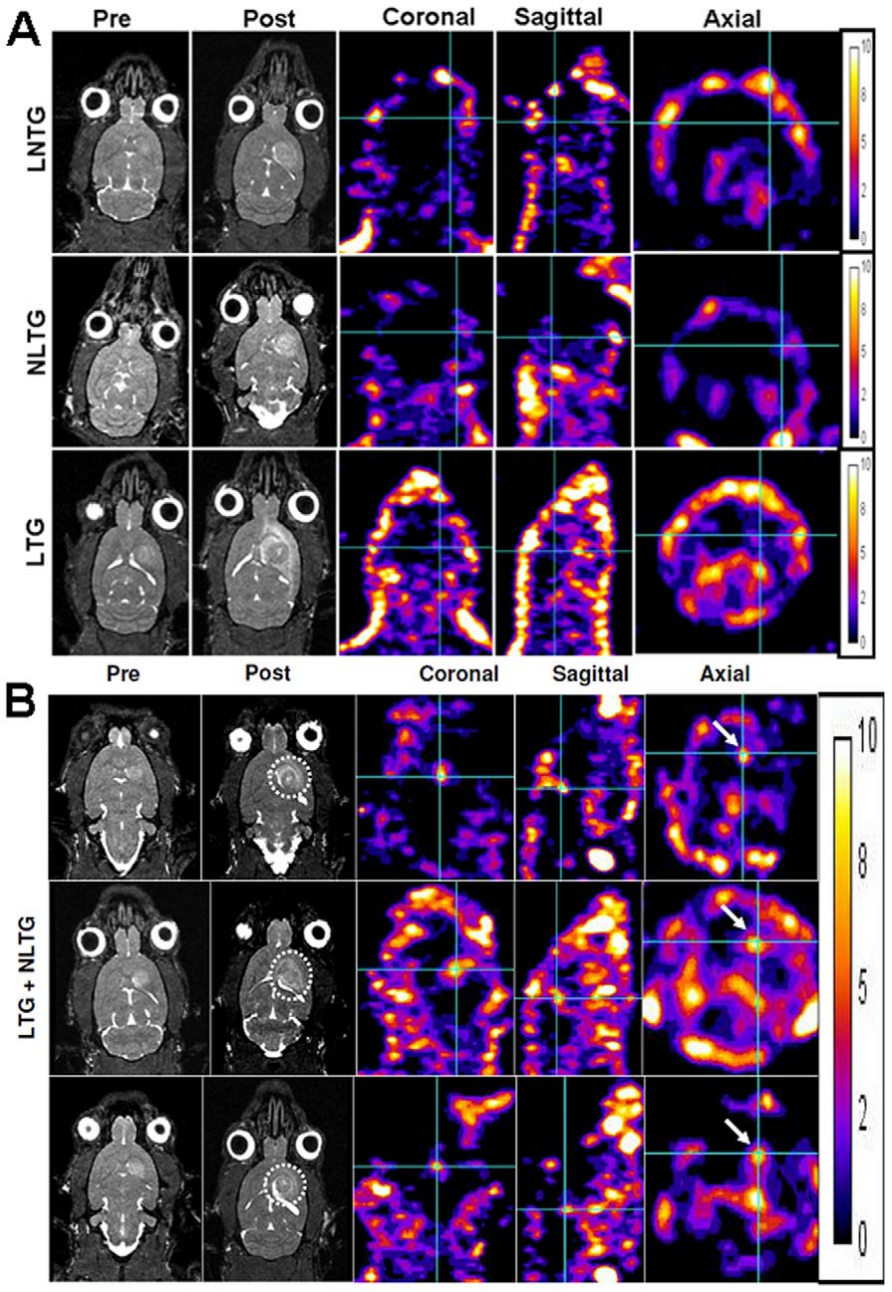
MRI and SPECT images for tracking of administered EPCs and gene expression. (**A**) **MRI and SPECT following administration of 5×10^6^ EPCs.** Pre-injection and 7 days post injection MR images of representative animals that received FePro labeled non-transduced (LNTG, upper row), non-FePro labeled transduced (NLTG, middle row) and FePro labeled transduced (LTG, lower row) EPCs intravenously. Note that low signal intensity areas/foci are seen in tumors that received FePro labeled cells (upper and lower rows, dotted circles) compared to the tumor that received non-FePro labeled cells (middle row). There was slight increase in focal activity of Tc-99m (white arrows) observed in tumor areas that received transduced EPCs (middle and lower rows). (**B**) **MRI and SPECT following administration of 10×10^6^ EPCs.** Pre-injection and 7 days post injection MR images of 3 representative animals that received 5 million FePro labeled transduced (LTG) plus 5 million non-FePro labeled, transduced (NLTG) EPCs. Note that low signal intensity areas/foci are seen in all tumors following administration of FePro labeled EPCs (dotted circles). There was increased focal activity of Tc-99m (white arrows) observed in all tumor areas.

**Figure 4:**
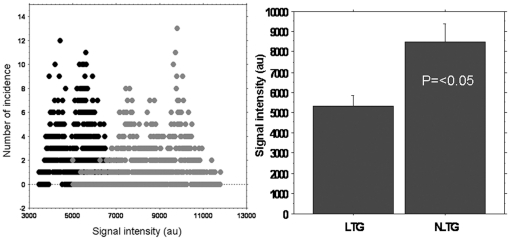
Histogram profile of signal intensity. Signal intensity histogram from representative animals of FePro labeled transduced (LTG) (black circles) and non-FePro labeled transduced (NLTG) groups (grey circles). Comparison of average signal intensity between two groups of animals showed significant differences (bar graphs). Note the low signal intensity in tumors that received FePro labeled transduced (LTG) cells (n = 3).

To determine the effect of transduction of exogenous gene (hNIS) on migration and tumor incorporation of transplanted EPCs, FePro labeled non-transduced EPCs, non-labeled transduced and FePro labeled transduced EPCs were intravenously administered to rats bearing U251 glioma that underwent MRI 1 day before and 7 days after EPCs' administration. As expected, all animals that received FePro labeled EPCs exhibited low signal intensity areas on MRI that was detected along the outer regions of the tumors, indicating accumulation of iron positive cells with no differences observed between the animals receiving FePro labeled non-transduced EPCs and FePro labeled hNIS transduced EPCs (**Figure 3A**). The accumulation of iron positive cells was confirmed by PB histochemical staining. Animals that received non-FePro labeled cells did not exhibit any significant low signal intensity areas in the tumors (**Figure 3A**). MRI analysis also showed significant differences (p<0.05) in signal intensity changes between the groups of animals that received FePro labeled and non-labeled transduced EPCs as shown by the histogram profiles of signal intensities (**Figure 4**).

**Figure 5:**
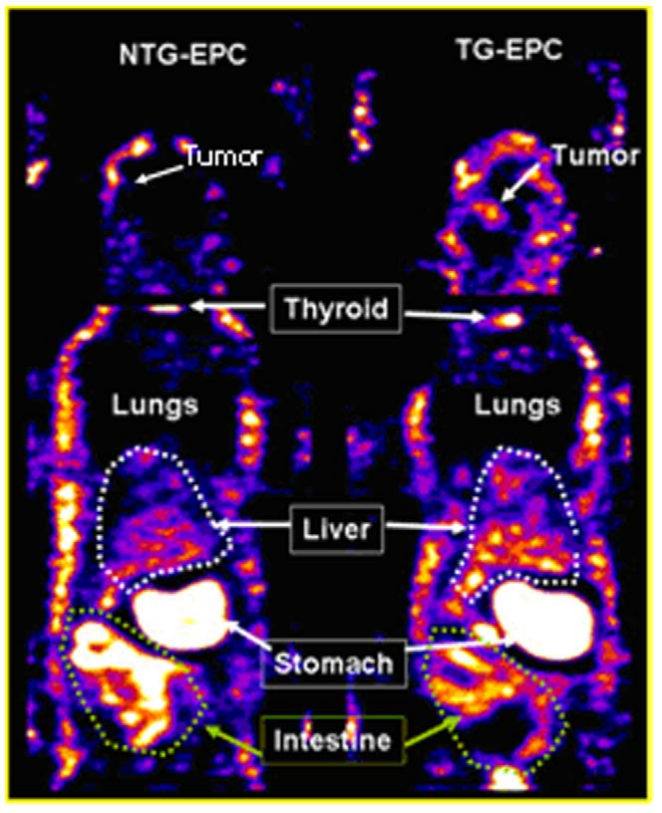
Whole body SPECT images for tracking of administered EPCs and gene expression. The distribution of Tc-99m activities is almost identical in animals bearing tumors that received non-transgenic (left) and transgenic EPCs, except the activities in the tumor and liver. Animal that received transgenic EPC showed higher activity in liver and tumor. Images are displayed in identical windows. Note there is no activity seen in the lungs and in the tumor that received non-transgenic EPCs (arrow, tumor).

**Figure 6:**
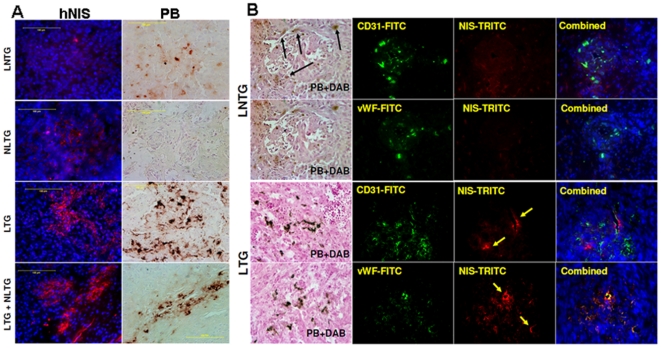
Cytochemistry of differentiated EPCs and Immunohistochemistry. (**A**) **hNIS and Prussian blue staining**: Immunohistochemistry of representative sections showed hNIS (red cells, left column) and iron positive cells (brown cells, right column) in FePro labeled non-transduced (LNTG), non-FePro labeled transduced (NLTG), FePro labeled transduced (LTG), and FePro labeled transduced plus non-FePro labeled transduced (LTG+NLTG) groups. Note that multiple iron positive cells are seen in tumors that received FePro labeled EPCs. hNIS positive cells are seen in tumors that received transduced EPCs. The animal that received FePro labeled non-transduced (LNTG) EPCs showed multiple iron positive cells but no hNIS positive cells (upper row, the small red dot is due to artifact). All tumors that received transduced cells showed multiple hNIS positive cells (red cells). The animals that received double dose of transduced EPCs (LTG+NLTG) showed more hNIS positive (red) cells than corresponding iron positive cells (brown). PB = DAB enhanced Prussian blue. (**B**) **Expression of human endothelial cell markers in the tumors**: Both FePro labeled non-transduced (LNTG) and FePro labeled transduced (LTG) administered EPCs showed incorporation into tumor vasculatures and expressed human specific endothelial markers (CD31 and vWF). Note the endothelial markers positive cells are also positive for hNIS indicating functional expression of transgenes.

To determine whether hNIS transduced EPCs expressed hNIS transgene product after migration and incorporation into the tumors, Tc-99m-SPECT images were obtained on day 8 after the administration of EPCs. It was expected that if hNIS protein was present in the tumor, Tc-99m activity would be higher compared to the tumor of the animals that received non-transduced EPCs due to the uptake of Tc-99m through trans-genetically encoded and expressed hNIS trans-membrane channel. Although MRI showed low signal intensity areas in animals that received 5×10^6^ of FePro labeled hNIS transduced cells, SPECT images showed only slightly increased Tc-99m activity, which was not seen in all animals (**Figure 3A**). Similarly the animals that received 5×10^6^ of non-FePro labeled hNIS transduced EPCs also showed slightly higher activity of Tc-99m in all tumor-bearing rats (**Figure 3A**). To increase the signal detected by SPECT, we have administered higher dose of hNIS transduced EPCs that included 10×10^6^ hNIS transduced cells out of which only 5×10^5^ cells were FePro labeled as well. This resulted in much higher activity of Tc-99m detected in rats that received double dose of transduced EPCs (**Figure 3B**) due to the higher number of cells expressing transgene hNIS protein channel. The activity was clearly visible within the tumor areas in all animals. **Figure 5** shows Tc-99m activities in different organs of representative glioma bearing animals that received control and hNIS transduced EPCs. Increased Tc-99m activity was seen in tumor and liver in animal that received hNIS transduced EPCs, indicating the expression of hNIS in the accumulated transduced EPCs. The activity in all other organs was similar between the two groups of animals (control EPCs vs. hNIS transduced EPCs). Immunohistochemistry confirmed hNIS positive cells in and around the tumors that received hNIS transduced EPCs and the number of detected hNIS positive cells were higher in rats that received double dose of hNIS transduced EPCs (10×10^6^ cells) compared to rats that received the dose of 5×10^6^ (**Figure 6A**). To determine whether iron labeling impaired the expression of hNIS transgene product in administered EPCs, same or consecutive tissue sections were stained with PB for iron positive cells. Tumors in animals that received both, FePro labeled and non-FePro labeled hNIS transduced EPCs showed multiple hNIS positive cells. However, many of the iron positive cells detected in the same area did not exhibit hNIS positivity (LGT, **Figure 6A**) indicating that not all of the administered FePro labeled hNIS transduced EPCs maintained the expression or hNIS protein after migrating to the tumor site.

### Expression of endothelial markers

To determine whether FePro labeled or unlabeled hNIS transduced EPCs incorporated into tumor angiogenesis or vasculogenesis, sections were double stained either with vWF and hNIS antibodies, or CD31 and hNIS antibodies. To confirm that the source of positive signals detected by these antibodies is from administered cells that were FePro labeled, consecutive tissue sections were stained with PB. In both cases multiple double positive cells were observed in the tumors (**Figure 6B**). The vWF and CD31 were also seen in tumors of the animals that received non-transduced EPCs.

### Distribution of administered EPCs

Supporting information S1 shows the distribution of IV administered In-111 labeled EPCs at different time points. About 40% of the injected cells distributed in the lungs at 1 hour, which cleared very rapidly and after 72 hours there was less than 2% of injected cells remaining. Activity in the liver and spleen remained stable from 2 to 72 hours. When normalized to tumor volume (cm^3^), there was average of more than 2.25% of injected EPCs that migrated to brain tumor, which indicate that about 112,500 EPCs accumulated (/cm^3^ of tumor mass) in glioma after 24 hours. On the other hand contralateral brain (CLB/cm3) showed minimum activity at 3 hours with a washout observed at 24 and 72 hours. The distribution pattern of In-111 labeled EPCs was different from that of In-111-oxine.

## Discussion

Our results proved our hypothesis that EPCs could be used to carry gene to the sites of interest and that the migration of EPCs carrying gene of interest can be tracked by *in vivo* imaging modalities. Low signal intensities detected by MRI enabled tracking of administered cells while increased uptake of Tc-99m in tumors, detected by *in vivo* SPECT, indicated functional expression of transgene after incorporation of EPCs in glioma. In that sense, EPCs can be used at the same time as probes for MRI or SPECT and as gene carrier systems.

### Importance of EPCs as gene carriers

Numerous previous publications, as well as our own reports, indicated the important role of cord blood or peripheral blood derived CD133^+^ EPCs in neo-vasculo- as well as angiogenesis associated with tumors or ischemic lesions [6], [18], [19]. Migration and homing of exogenously administered or bone marrow mobilized endogenous EPCs to tumor or ischemic lesions are dependent on the expression of stromal derived factor (SDF-1) [6], [20], [21]. SDF-1 is chemo-attractant for cells such as EPCs, that express abundant CXCR4 receptors [21] and our previous work demonstrated that exogenously administered, either peripheral blood derived CD133^+^ or cord blood derived adult CD133^+^ EPCs, targeted and migrated to the areas of tumors where HIF-1 mediated SDF-1 expression was significantly increased [6]. Here we show that as expected, transduced EPCs carrying hNIS gene migrated and homed to the sites of tumors. Immunohistochemistry also indicated that these EPCs migrated and incorporated into tumor angiogenesis/vasculogenesis that was in accordance with now well established evidence that EPCs are involved in both vasculogenic and angiogenic processes [18], [20], [22], [23]. Transduced EPCs also expressed functional gene product that resulted in intracellular uptake of Tc-99m through trans-genetically expressed hNIS channel which was detected by *in vivo* SPECT. In addition, hNIS expression was confirmed by immunohistochemistry. Expression of functional product by exogenously introduced gene is extremely important and strongly depends on the promoter used in driving that expression [17]. Previously we have demonstrated that when viral vectors were used, EF-1 and CMV promoters were very effective in driving the expression of exogenous genes. Here, we have selected to use CMV as promoter because of demonstrated sustained long-term expression of transgene in the cells [17]. Based on our successful transduction and use of EPCs to carry the gene to the sites of tumor, future experiments can be designed to develop therapeutic strategies. Here we used hNIS as reporter protein to be detected by Tc-99m SPECT, however, expression of hNIS can also be utilized to induce cell death as radioactive iodine (such as I-131) is actively taken up by the transduced cells. In addition, hNIS can also act as check-and-balance mechanism, where I-131 can be used to control unwanted growth of transduced cells. Therefore, hNIS can be used both, as an *in vivo* reporter and as a suicidal gene. Alternatively, EPCs can be used as gene carriers for other proteins with anti-tumor effects. For example, EPCs can be transduced with either double suicidal genes, such as Cytosine Deaminase/herpes simplex virus-1 Thymidine Kinase (CDTK) [24], [25], or genes that can be used to express inflammatory or cytotoxic cytokines [26], [27] or cytochrome P4502B1 (CYP2B1) gene that is to increase intratumoral concentrations of pro-drugs [28]. In addition, blockage of vascular endothelial growth factor (VEGF) function using soluble vascular endothelial growth factor receptor 1 gene can be used to inhibit tumor growth [29], [30]. Alternatively, immune agents such as Interferon-beta (IFN-beta) genes can also be delivered using EPCs to suppress the tumor growth [31]. However, to prevent non-specific expression of suicidal gene products in transduced cells in different unwanted targets, vector can be designed for conditional expression of transgene product using tissue specific promoters.

### EPCs as probes for MRI and SPECT

FePro labeling allows for EPCs' migration to be tracked by *in vivo* MRI to homing sites, implanted glioma in rats in this study. Previously, we have reported tracking of locally or intravenously administered EPCs detected by high strength small animal MR scanner to the sites of active angiogenesis in implanted subcutaneous and intracranial tumors [5], [6], [32]. In this study, we have used clinical 3 Tesla MRI systems to track intravenously administered cord blood derived EPCs to intracranial glioma tumor where administered EPCs exhibited accumulation at the sites of active angiogenesis. All animals that received 5×10^6^ of FePro labeled transduced or non-transduced EPCs showed areas of low signal intensity in and around the tumor 7 days following administration of cells. However, SPECT analysis showed a slight increase in Tc-99m activity in tumors that received 5×10^6^ of either FePro labeled or unlabeled transduced EPCs. But, when the number of administered cells was increased to 10×10^6^ cells, increase in Tc-99m activity was detected by SPECT in tumors of all animals. These findings indicated that transplanted cell accumulation within the tumor at the administered dose of 5 million cells, did not result in accumulation of sufficient amounts of Tc-99m to be detected by SPECT scanning. On the other hand, at the same dose of 5 million cells tumor accumulation of FePro labeled transduced and non-transduced EPCs exhibited significant change in low intensity signals detected by MRI. Based on our previously reported FePro labeling method that ensured for almost 100% labeling efficiency [8], it was expected that at the dose of 5 million FePro labeled cells, the migrated cells would carry the iron to the sites of interests and the number would be sufficient to be detected by MRI. However, the same was not observed on SPECT analysis with hNIS FePro labeled or non-labeled transduced cells. The number of cells expressing functional hNIS that migrated to the tumor was at least 2 times less (40% transduction efficiency) than that of cells that contained MRI detectable levels of FePro, and that was apparently below the detection Tc-99m threshold of SPECT. Since transduction efficiency was not more than 40% and the selection of transduced cells was not performed prior to transplantation it was expected that increasing the dose of administered cells would reach this threshold. When the number of administered transduced cells was increased to 10×10^6^, there was clear visualization of Tc-99m activity in the tumors. Under circumstances of achieving almost 100% of transduction efficiency, both MRI and SPECT could be considered to have exhibited similar sensitivity in detecting migrated EPCs in the tumors. Still, it is to note that iron labeling is much more efficient than lentivirus transduction and therefore practical sensitivity of MRI is greater. However, hNIS gene transduction in conjunction with SPECT analysis was used here as a proof of principle study. When developing therapeutic and/or diagnostic approaches the choice of gene may not be linked with imaging detection, however MRI could still be used for the purpose of tracking and monitoring the fate of transplanted gene transduced cells. Current methods of MRI cannot estimate the number of incorporated magnetically labeled cells in area of interest due to endogenous iron, hemorrhage in the tumors and slower blood flow in the tumors. All of these will show low signal intensity areas on T2*-weighted or FIESTA images as we have previously reported [33]. However, if necessary to determine the numbers of migrated cells, EPCs can be tagged with In-111 or Tc-99m-HMPAO before injection. Our supplemental studies (supporting information S1) showed that 2.25% of administered In-111 labeled EPCs accumulated in tumor, which indicated the accumulation of 112,500 EPCs in the tumor. Terrovitis et al [34] have indicated that accumulation of 1×10^5^ of transduced cells carrying hNIS are needed to be detected *in vivo* by Tc-99m SPECT. Based on this value and considering our transduction efficiency, at least 2% of the administered cells migrated to the sites of tumors, which is also proven by In-111 labeled EPCs studies.

Following IV injection, EPCs will pass through liver and spleen where some of the administered cells may be held up. In addition, some of the administered EPCs will also home to bone marrow. During this migratory process all the injected EPCs that have died are expected to be cleared by liver and spleen. Only active and viable EPCs will migrate and accumulate at the margin of the tumor due to the specific migratory signals, such as HIF-1α induced SDF-1 expression [6], [35]. Once at the tumor site where vasculogenesis is the most active, injected EPCs will either be incorporated into the tumor neovasculatures or remain idle without incorporating into the angiogenic processes. However, further investigations are warranted to determine the minimal number of hNIS transduced cells needed to be detected by Tc-99m SPECT at the site of tumors or other lesions following IV administration.

### Tracking of hNIS positive transduced stem cells and advantage of SPECT

Investigators have used transduced stem cells and tracked them by various *in vivo* imaging modalities. However, most of investigations dealt with local administration of transduced cells followed by *in vivo* imaging of the gene product by bioluminescence, fluorescence, SPECT or PET scanning [36], [37], [38]. Tracking of intravenously administered transduced cells by nuclear medicine techniques is challenging and requires sensitive imaging modalities, stable transduction and use of radionuclide that will be optimal in respect to energy, half-life and cost. PET is very sensitive imaging modality and has been widely used to track systemically administered genetically transduced cells. One of the most widely used reporter genes for PET imaging is wild-type herpes simplex virus type 1 thymidine kinase (HSV1-tk) and it's HSV1-sr39tk mutant. This enzyme efficiently phosphorylates purine and pyrimidine analogs and has been very successfully used with radio labeled reporter probes such as ^124^I-2′-fluoro-2′-deoxy-1-β-D-β-arabinofuranosyl-5-iodouracil (FIAU), ^18^F-2′-fluoro-2′-deoxy-1-β-D-β-arabinofuranosyl-5-ethyluracil (FEAU) and ^18^F-9-(4- ^18^F-fluoro-3-hydroxymethyl-butyl)guanine (FHBG) [39], [40], [41], [42]. hNIS positive cells can also be tracked with I-124, a positron emitter [43]. However, the disadvantage of PET is its short half-lived radionuclides with higher energy (511 keV) and repeated scanning with high energy tracer could be detrimental to the transduced cells. In addition dedicated radiochemistry laboratory and cyclotron are needed, which are very expensive. On the other hand, although less sensitive than PET, SPECT can utilize relatively short half-lived and low energy radionuclides, such as Tc-99m and I-123, to track hNIS transduced cells. These radiotracers are available through vendors and are very cheap compared to PET tracers. Moreover, PET and dedicated radiochemistry laboratory are not widely available to molecular imaging investigators.

### Conclusion

This is the first study to report the use of cord blood derived EPCs to carry a gene (hNIS) to the sites of glioma, and the migration and the expression of gene products were determined by *in vivo* MRI and SPECT studies, respectively. These EPCs were used both as gene carrier and imaging probes. EPCs can be used to deliver therapeutic genes to the sites of lesions. The use of EPCs to carry therapeutic gene to the sites of different lesions are underway in our laboratory.

## Supporting Information

Supporting Information S1 To determine the distribution of IV administered EPCs to different organs in tumor bearing rats, In-111-oxine labeled EPCs were administered IV on day 21 following tumor implantation. Cells were labeled with In-111-oxine using a standard method keeping the In-111 dose at 2–3 bq/cell. SPECT images were obtained at different time points, from 1 hour to 72 hours. To differentiate between the activity of In-111 and that of In-111 labeled cells on SPECT images, animals bearing glioma that received In-111-oxine alone also underwent SPECT images were obtained at different time points. Supporting information **Figures S1 and S2** show the biodistribution of In-111-oxine and In-111 labeled EPCs in different organs and in the tumors.
(DOC)
