# Supplementary material for: Endothelial Progenitor Cells (EPCs) as Gene Carrier System for Rat Model of Human Glioma
Source: PLoS One. 2012 Jan 20;7(1):e30310. doi: 10.1371/journal.pone.0030310 (PMC3262815; doi:10.1371/journal.pone.0030310)
Supplement: Supporting Information S1 — To determine the distribution of IV administered EPCs to different organs in tumor bearing rats, In-111-oxine labeled EPCs were administered IV on day 21 following tumor implantation. Cells were labeled with In-111-oxine using a standard method keeping the In-111 dose at 2–3 bq/cell. SPECT images were obtained at different time points, from 1 hour to 72 hours. To differentiate between the activity of In-111 and that of In-111 labeled cells on SPECT images, animals bearing glioma that received In-111-oxine alone also underwent SPECT images were obtained at different time points. Supporting information Figures S1 and S2 show the biodistribution of In-111-oxine and In-111 labeled EPCs in different organs and in the tumors. (DOC) [file pone.0030310.s001.doc]

**Title:** Endothelial progenitor cells (EPCs) as gene carrier system for rat model of human glioma

**Authors:** Nadimpalli Ravi S Varma, Branislava Janic, ASM Iskander, Adarsh Shankar, Mohammed P.I. Bhuiyan, Hamid Soltanian-Zadeh, Quan Jiang, Kenneth Barton, Meser M. Ali, Ali S. Arbab.

**Supplemental studies:** To determine the distribution of IV administered EPCs to different organs in tumor bearing rats, In-111-oxine labeled EPCs were administered IV on day 21 following tumor implantation. Cells were labeled with In-111-oxine using a standard method keeping the In-111 dose at 2-3 bq/cell. SPECT images were obtained at different time points, from 1 hour to 72 hours. To differentiate between the activity of In-111 and that of In-111 labeled cells on SPECT images, animals bearing glioma that received In-111-oxine alone also underwent SPECT images were obtained at different time points. Supporting information **Figures S1 and S2** show the biodistribution of In-111-oxine and In-111 labeled EPCs in different organs and in the tumors.

**Figure S1**: **Biodistribution of In-111-oxine and In-111 labeled EPCs**

Biodistribution of administered In-111-oxine (upper 2 panels) and In-111 labeled EPCs (5x10^6^ EPCs, lower 2 panels) in whole body (WB) and in implanted glioma from 1 hour to 72 hour. Unlike popular belief that IV administered cells remain in the lungs for extended time, majority of EPCs cleared from lungs within 3 hours and very low activity seen after 72 hours. The WB distribution patterns of In-111-oxine and In-111 labeled EPCs were different. Distribution to the glioma was also different. Note the increased accumulation of In-111 labeled EPCs in glioma after 24 hours, whereas non-specific In-111-oxine activity clears from the tumor over time (yellow circles). Inset in upper panel shows contrast enhanced glioma on MRI (yellow arrows).


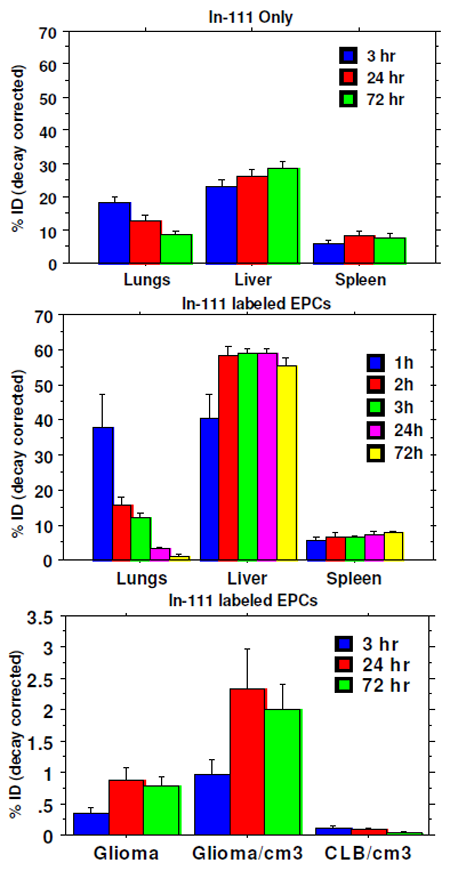


**Figure S2:** **Semi-quantitative analysis**

The bar graphs represent n= 5 (In-111-oxine) to 9 (In-111 labeled EPCs) animals. Data is expressed decay corrected percent injected dose (%ID). Note there is very low %ID of EPCs remaining in the lungs at 72 hour. When normalized to tumor volume (/cm^3^), there is average of more than 2.25% injected EPCs migrated to brain tumor, which indicate about 112,500 EPCs accumulated (/cm^3^ of tumor mass) in glioma on 24 hours. On the other hand contralateral brain (CLB/cm3) showed minimum activity at 3 hours with washout at 24 and 72 hours.
